# An Echo of the Nineteenth Century

**Published:** 1920-10-16

**Authors:** 


### An Echo of the Nineteenth Century.

On March 30 last there died at Bishops Waltham Dame Adela Lucy Leman Jenner at the age of seventy-five. At the time very small reference was made to the death of Lady Jenner, who was the widow of Sir William Jenner, Physician in Ordinary to Queen Victoria, and later to the Prince of Wales (King Edward VII.). Lady Jenner survived her husband by twenty-two years, and their joint lives covered a period of 105 years—that is, from 1815, the year of Waterloo, until two years after the Great European War.

It will be remembered that the most notable work of Sir William Jenner was the investigations which enabled him practically to prove the difference between typhoid and typhus fevers. He attended the Prince Consort in his last illness, and he is several times mentioned in the letters of Queen Victoria to her uncle, the King of the Belgians. Writing to him on December 12, 1861, the Queen said: "I cannot sufficiently praise the skill. attention, and devotion of Dr. Jenner. He is the first fever doctor in Europe, one may say." On that date the condition of her husband was considered to be favourable, and the letter was full of hope. The illustrious patient, however, died on the 14th instant, and on the 20th, when the Queen again wrote to her uncle, she refers to "our excellent Dr. Jenner, who has been, and is, my great comfort."

Many allusions to Dr. Jenner that one comes across in works of reference are in connection with fever. First we have his investigations of typhus and typhoid, next his treatment of the Prince Consort for fever, and again his attendance in 1871 upon the Prince of Wales during the attack of typhoid which so nearly ended fatally.

It is not surprising that Lady Jenner, with all her association with the Royal Family, should have been in possession of many mementoes of the Court. Under her will, particulars of which have just been published, she leaves the portrait of her husband by Frank Holl, R.A., and letters from Queen Victoria to her husband and herself to her son Sir Walter Jenner, desiring that they shall devolve as heirlooms with the baronetcy. She also leaves him a ring given to her husband by the Duc de Montpensier, three pieces of plate given by Queen Victoria, and a clock given to her husband by the Duke of Connaught to her son Albert, and articles given to herself and Sir William Jenner by other members of the Royal Family to her son Leopold.

